# Basic life support knowledge, self-reported skills and fears in Danish high school students and effect of a single 45-min training session run by junior doctors; a prospective cohort study

**DOI:** 10.1186/1757-7241-22-24

**Published:** 2014-04-14

**Authors:** Anne Marie Roust Aaberg, Caroline Emilie Brenner Larsen, Bodil Steen Rasmussen, Carolina Malta Hansen, Jacob Moesgaaard Larsen

**Affiliations:** 1 Department of Anesthesia and Intensive Care Medicine, Aalborg University Hospital, Hobrovej 18-22, Aalborg 9000, Denmark; 2 Department of Cardiology and Center for Cardiovascular Research, Aalborg University Hospital, Hobrovej 18-22, Aalborg 9000, Denmark; 3 Department of Cardiology, Gentofte Hospital, Niels Andersens Vej 65, post 635, Hellerup 2900, Denmark

**Keywords:** MeSH terms: Heart arrest, Out-of-hospital cardiac arrest, Basic life support, Cardio pulmonary resuscitation, Automated external defibrillation, Youth, Education

## Abstract

**Background:**

Early recognition and immediate bystander cardiopulmonary resuscitation are critical determinants of survival after out-of-hospital cardiac arrest (OHCA). Our aim was to evaluate current knowledge on basic life support (BLS) in Danish high school students and benefits of a single training session run by junior doctors.

**Methods:**

Six-hundred-fifty-one students were included. They underwent one 45-minute BLS training session including theoretical aspects and hands-on training with mannequins. The students completed a baseline questionnaire before the training session and a follow-up questionnaire one week later. The questionnaire consisted of an eight item multiple-choice test on BLS knowledge, a four-level evaluation of self-assessed BLS skills and evaluation of fear based on a qualitative description and visual analog scale from 0 to 10 for being first responder.

**Results:**

Sixty-three percent of the students (413/651) had participated in prior BLS training. Only 28% (179/651) knew how to correctly recognize normal breathing. The majority was afraid of exacerbating the condition or causing death by intervening as first responder. The response rate at follow-up was 61% (399/651). There was a significant improvement in correct answers on the multiple-choice test (p < .001). The proportion of students feeling well prepared to perform BLS increased from 30% to 90% (p < .001), and the level of fear of being first responder was decreased 6.8 ± 2.2 to 5.5 ± 2.4 (p < .001).

**Conclusion:**

Knowledge of key areas of BLS is poor among high school students. One hands-on training session run by junior doctors seems to be efficient to empower the students to be first responders to OHCA.

## Introduction

Early recognition, performance of cardiopulmonary resuscitation (CPR) and the immediate activation of emergency medical services (EMS) are critical determinants of survival after out-of-hospital-cardiac-arrest (OCHA) [1].

Recent data from the Danish OHCA registry shows an improvement in bystander CPR increasing from 21% of cases in 2001 to 45% in 2010, with a concomitant improvement in overall 30-day survival from 4% in 2001 to 11% in 2010 [2]. However, the majority of OHCA still receive no bystander CPR, especially in non-public areas where most OHCA occur thus contributing to the low survival due to delayed or no bystander CPR [3,4]. Bystanders with previous CPR training are more likely to perform CPR [5]. Accordingly, the International Liaison Committee on Resuscitation and the American Heart Association (AHA) recommend that CPR training should be implemented throughout the community and be incorporated as a standard part of the school curriculum [6].

Over the past years several methods to teach school children have been developed and tested, showing that BLS training is effective in children from age of 4 years. However, the most efficient method is not well established [7]. Previous studies have shown that training should start at an early age, be repeated at regular intervals and be hands-on oriented because children only receiving theoretical training perform poorly [8]. In spite of this current knowledge, there is no consensus as to which method or material should be used to train students in BLS [6,9]. Additional barriers to implementing BLS in schools are limited resources and time in the curriculum [6].

The aim of the study was to evaluate Danish high school students’ current BLS knowledge, and the effect of a single 45-minute BLS hands-on training session run by junior doctors on theoretical knowledge, self-assessed skills and self-perceived fears related to performing BLS.

## Methods

### Study design and participants

This is a prospective cohort study conducted in October 2012. Six-hundred-fifty-one students were included regardless of their previous attendance of a CPR course. They represented all three high school levels (first to third year) at the public Cathedral High School in Aalborg, the fourth largest city in Denmark. The study participation was voluntary and no student declined to participate at baseline.

### The questionnaire

The students answered an identical questionnaire immediately before and one week after the training session. The questionnaire incorporated history of prior BLS training, multiple-choice questions on BLS theory (item 1-8), self-assessed skills (item 9) and self-perceived fear of being first responder to a person with OHCA (item 10). Item 9 was evaluated on a 4-point scale of “Not able to perform BLS”, “In doubt and would probably not help”,”Know the theory but have no practical skills” and “Well prepared and would take action”. Item 10 was evaluated on a visual analogue scale from zero (no fear) to ten (the worst imaginable fear). A supplementary qualitative description in a single sentence of their worst fear was encouraged. The questionnaire is available as supplementary online material in an English version translated using an English- Danish correspondent, from the original Danish version (Additional file 1).

The content of the questionnaire was validated through cognitive interview of 10 lay persons to ensure that each item was understandable and the answers unambiguous. Test-retest reliability was examined in 32 students from another high school in the area, who did not receive the BLS training session, using two questionnaires at one week interval. No significant differences in any of the above items were observed.

### The BLS training and data collection

The instructors were four doctors aged 27 to 30 years with less than one year of clinical experience since graduation from medical school. Generic teaching experience varied among the instructors but none had prior BLS teaching experience. All the junior doctors underwent the same training course in acute medicine within the last 1 ½ year of medical school, led by Aalborg University Hospital. The course comprises advanced life support training in accordance with current guidelines by the European Resuscitation Council.

The training course for the high school students in this study was developed by two of the authors (ARA, CBL) based on a short protocol, in accordance with the ERC Guidelines for Resuscitation 2010. The students were trained in groups of 40-60 students giving a student-to-instructor ratio that did not exceed 15:1. Each training session lasted 45 minutes and included theoretical aspects of BLS and hands-on training using mannequins. The students were divided into subgroups circulating between three skill stations with the themes “Breathing assessment”, “Recognition of a cardiac arrest” and “How to perform CPR”. An introduction to the automatic external defibrillator (AED) was given in one big group at the end of the lesson.

### Statistical analysis

The statistical analysis was performed using Stata 11.2 (StataCorp, College Station Texas, USA). P-value < .05 was considered statistically significant. Missing values in questionnaires of responders were imputed using multiple imputations by sex, age, previous BLS training and the student answers in the rest of the questionnaire. Responders and non-responders at follow-up were compared using non-paired *t*-test and chi2-test. For the responder sub-cohort a change over time in continuous variables was tested using paired t-tests and change in dichotomous variables was tested using conditional logistic regression.

## Results

### Demographics

All 651 students answered the baseline pretest questionnaire. Age ranged from 17 to 21 years, 17.5 ± 1.2 years (mean ± SD). Sixty-eight percent were women. Sixty-three percent (413/651) of the students had received prior BLS training either in primary school, driving schools or sport clubs. The follow-up questionnaire was completed by 399 students (response rate 61%). There were no significant differences at baseline between responders and non-responders at follow-up regarding age, sex, prior BLS training, knowledge concerning BLS training (question 1-8), the changes in self-assessed BLS-skills and level of self-perceived fear of being first responder to a cardiac arrest situation.

### Theoretical knowledge

The proportions of correct answers to the multiple-choice items at baseline are shown in Table 1. Ninety-nine percent of all the students knew how to call EMS in case of a cardiac arrest, 28% knew how to evaluate whether an unconscious person has adequate breathing and 57% knew what to do in a situation where the level of unconsciousness is uncertain. Sixty-six percent of the students knew the correct 30:2 compression-ventilation ratio during CPR.

**Table 1:** Participant characteristics

**Baseline population n = 651**	**Cathedral high school**	**Missing (%)**	**Odds ratio**	**95% CI**	**p-value**
Age (mean ± sd)	17.5 ± 1.2	0			
Women n (%)	441 (67.7)	0			
Previous BLS training n (%)	413 (63.4)	0			
Correct answers baseline n (%)			Correct multiple choice answers one week after BLS training		
What is a heart attack?	461 (70.8)	3.7	13.9	(6.4;30.8)	>0.001
Suddenly a person becomes consciousness and falls over. What do you do?	394 (60.5)	4.8	1.9	(1.4;2.7)	>0.001
How do you know if a person is breathing normally?	179 (27.5)	3.8	3.2	(2.2;4.6)	>0.001
Who do you call in a heart attack situation?	644 (98.9)	0.5	0.7	(0.1;4.0)	0.66
What is basic life support?	429 (65.9)	2	123.3	(17.2;884.4)	>0.001
How do you perform artificial ventilation to an unconscious person?	580 (89.1)	1.7	8.7	(3.1;24.4)	>0.001
What do you do if you are uncertain if a person is unconscious?	369 (56.9)	5.7	119.6	(16.2;884.7)	>0.001
What is a automated external defibrillator (AED)	593 (91.1)	2.7	6.2	(2.1;17.9)	>0.001

Basic life support questiones item 1-8. Proportion (%) of correct answers to each question at baseline and percentage of missing answers. Odds ratio, CI and p < value for correct multiple choice answers one week after BLS training.

The odds ratios for the correct answer at follow-up are shown in Table 1. There were significant improvements for all but one multiple-choice item (p < .001); i.e. item 4, who to call in case of a sudden loss of consciousness (p = .66). This item was already correctly answered at baseline by 99% of the students.

### Self-assed BLS skills

**Figure 1:**
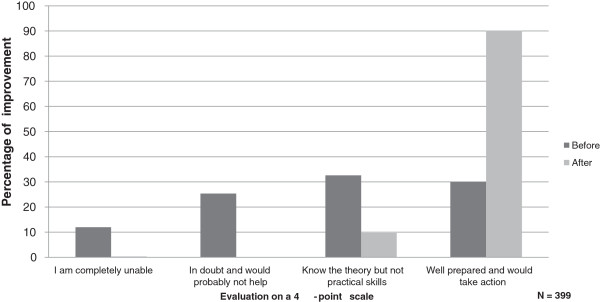
**Self-assessed skills.** Proportion (%) of students expressing an improvement of their confidence in own skills, before and after the training session.

The figure illustrates the changes in self-assessed BLS skills (item 9) (Figure 1). At baseline approximately one third of the students answered that they would not respond or were uncertain about how to respond in case of OHCA, but at follow-up 90% felt well prepared and would take action (p < .001).

### Self-perceived fear of being first responder to a cardiac arrest situation

The level of fear of being a first responder to a person having cardiac arrest was significantly decreased from baseline to follow-up with a decline in visual analog scale-score from 6.8 ± 2.2 to 5.5 ± 2.4 (p < .001). The supplementary qualitative description of their worst fear at baseline had three common themes: Fear of doing something wrong, fear of being the cause of exacerbating the situation and fear of being the cause of the person’s death. The most common sentence reflecting their fear at baseline was the thought of panic. Only a minority of the answers, showed that the students were afraid of being accused of murder and feeling guilty if “things go wrong”. Most of the students were afraid of misjudging the situation and of providing incorrect and inappropriate assistance. At follow-up the themes were similar to those at baseline. Furthermore the students still mentioned at numerous occasions that they feared the person would die.

## Discussion

The main findings from this study of high school students´ BLS knowledge, skills and fears were that most high school students lacked knowledge regarding the first three steps of the chain of survival, had poor self-assessed BLS skills and high self-perceived fear of being first responder, despite previous training. Notably, one 45-minute training session based on ERC guidelines, consisting of a hands-on training session and a short theoretical introduction run by junior doctors, had an impact on empowering the students as first responders to a cardiac arrest. This shows that even though the students had divergent a priori qualifications, likely to be the case in real world settings, they increased their knowledge and self-assessed skills.

### Theoretical knowledge and interventions on BLS in high school students

It is noteworthy that a significant proportion of students lacked knowledge regarding the first three links in the chain of survival despite previous training. Although our study was not designed to assess qualitative aspects of students’ previous training and how these affected their current knowledge, our results are in accordance with previous findings that retaining CPR skills requires repetitive training [7].

### Self-assessed BLS skills and self-perceived fears of being first responder

The high school students’ self-assessed BLS skills and self-perceived capability to take action as the first responder increased significantly in the present study. There is an inconsistency concerning self-confidence to perform CPR among high school students in similar studies. Meissner et al. found that only 27% of the high school students dared to initiate CPR before the training session which increased to 99% after a four hour training day [8]. Parnell found that 84% of the students would be willing to perform CPR on a family member and 64% on a stranger. The findings of a Norwegian study were that despite a good theoretical knowledge about handling a lifeless adult, self-reported confidence in having sufficient BLS knowledge was only modest [10].

The fears of being first responder in a cardiac arrest situation observed in this study indicate common themes of causes for not providing bystander CPR as identified in other studies [11]. Unfortunately, our study was not designed to address fear as an independent topic, since fear was not a topic in the training course. Even though there was a change in the level of fear of out participants, many participants still reported the same themes of fear after the course. However, a more thorough analysis of our qualitative question was not possible due to the study design. We acknowledge that it is an important aspect and that future studies should be designed to investigate how to decrease fear among high school students by including a brief discussion about the student´s fears concerning basic life support in BLS courses.

### Teaching high school students as a strategy to reach a broad audience

Even though bystander CPR is a well-established determinant of survival after OHCA, the proportion of cardiac arrest victims who receive bystander CPR remains low, especially after OHCA at home [2]. Both acquisition of skills and skill retention after conventional CPR training have been disappointing and in this context, training of children and young adults is valuable for three main reasons. First, as a long-term investment, since training the young will eventually lead to a whole generation of trained adults, as reported in Stavanger [12]. Secondly, schoolchildren are at an age when knowledge and skills are well retained [13]. Thirdly, although they account for only a small percentage of OHCA, children/young adults should be capable of being first responder at OHCA as the majority occurs in private homes. If the students share the acquired knowledge with their family and non-trained friends, it indirectly introduces the BLS training to a broader audience, as previously shown [8]. Finally, the positive attitude towards learning BLS among high school students [14] could be a critical determinant of the impact of these short training sessions and emphasizes the importance to cease this golden opportunity to increase the knowledge on BLS at an early time in life.

### Implementation of BLS in Schools

Even though the AHA and recommends CPR training of schoolchildren to be mandatory, implementation and maintenance of teaching BLS within the school system is a challenge worldwide [6]. Several key aspects to achieve successful implementation remain unknown, such as the length and the repetitive sequence of training sessions, the content, the teacher (professional instructor vs. non-professional instructor) and the methods or materials that should be used [15-17]. Previous studies have identified cost, limited time in the curriculum and instructor scheduling difficulties as the main barriers to implementation of BLS in schools [6,18]. Our results are valuable in this context, since our study used a new method where voluntary junior doctors used standard ERC BLS guidelines to assess students´ level of knowledge and skills and to carry out an efficient 45-minute hands-on training session and achieve considerable increase in knowledge, self-assessed skills and significant decrease in self-perceived fear. Further, the short time frame used in our study (one 45-minute lesson) is a more realistic time frame to include in school curriculums compared to previous studies of 4-hour session [19]. Collectively, our results propose a new, simple and feasible method to teach BLS in schools which is shorter and cheaper than previous hands-on studies and could more easily be repeated once a year [8,19].

### Study limitations

In the present study, the follow-up questionnaire was handed out one week after baseline. The long-term effect of a single 45-minute training session on high school students therefore cannot be evaluated in this study. Furthermore we did not evaluate the quality of the educational skills of the instructors. However, we only used junior doctors who were trained using the same protocol based on ERC´s guidelines.

There is some risk of selection bias because we only examined one high-school, but for logistical reasons this were the only option. Aalborg Cathedral School represents a broad sample of students because the students attending are enrolled throughout the whole region of northern Denmark including both rural areas and the city. And since this study represents a broad sample of students we believe it provides a god insight. Moreover the responders and non-responders were comparable in age, gender, education level (high school) prior BLS training, knowledge concerning BLS, self-assed skills and self-perceived fear of being first responder to a person with OHCA.

Even though all the students enrolled on a voluntary base the dropout was 39% (254/651) after one week follow-up. Some of the reasons being; the students’ skipped class, disease among the students, confusion regarding the teachers when to hand out the follow-up and where to hand the questionnaires in.

## Conclusion

The BLS knowledge among high school students is poor, despite previous training. One 45-minute hands-on session run by junior doctors seems to be efficient to increase BLS knowledge and empower high school students to act as first responders in case of cardiac arrest on a short term- scale.

## Abbreviations

OHCA: Out-of-hospital cardiac arrest; BLS: Basic life support; CPR: Cardio pulmonary resuscitation; EMS: Emergency medical services; AHA: American Heart Association; ERC: European Resuscitation Council; AED: Automated external defibrillator.

## Competing interests

The authors declare that they have no competing interests.

## Authors’ contributions

AAMR and LCB contributed to all parts of the study i.e. conception of the idea, construction of the questionnaire, data collection, data analysis and writing the manuscript. RBS and LJM contributed to construction of the questionnaire, data analysis and writing the manuscript. HCM contributed to data analysis and writing the manuscript. The statistical analysis was made in collaboration with senior biostatisticians. All authors read and approved the final manuscript.

## Supplementary Material

Additional file 1 The questionnaire in an English version translated using an English-Danish correspondent, from the original Danish version. Click here for file
