# Supplementary material for: Basic life support knowledge, self-reported skills and fears in Danish high school students and effect of a single 45-min training session run by junior doctors; a prospective cohort study
Source: Scand J Trauma Resusc Emerg Med. 2014 Apr 14;22:24. doi: 10.1186/1757-7241-22-24 (PMC4022325; doi:10.1186/1757-7241-22-24)
Supplement: Additional file 1 — The questionnaire in an English version translated using an English-Danish correspondent, from the original Danish version. [file 1757-7241-22-24-S1.pdf]

Full name ______________________ □ **Male** □ **Female**

Age and date of birth ______________________

Have you received prior training in basic life support?

If yes, when and where ____________________________________

(Tick the answer you think is most appropriate. Only tick one box for each question.)

1: What is a cardiac arrest?

□ The body is taking a break
□ A cardiac arrest means that the person is dead and nothing more can be done
□ Cardiac arrest is something only doctors can treat
□ The person is unconscious and not breathing normally (correct)

2: Suddenly a person becomes unconscious and collapses. What do you do?

□ Nothing
□ Call for help and then shake the person to see if he/she wakes up (correct)
□ Shake the person hard to see if he/she wakes up, and then wait for help
□ Start CPR

3: How do you know if a person is breathing normally?

□ If I hear gasping, slow or strained breathing, I must assume that the person is breathing
□ To determine whether the person is breathing normally I have to clear the airway
□ As long as the person is not looking completely blue or strange, I assume that he/she is breathing
□ I put my ear over the mouth looking for the chest to rise

4: Who do you call in a cardiac arrest situation?

□ My parents
□ The Emergency Medical **112** (Correct)
□ The police **114**
□ A doctor

5: What is basic life support?

□ 40 chest compressions in the middle of the chest followed by 2 ventilations
□ 30 chest compressions in the middle of the chest followed by 2 ventilations (correct)
□ 20 chest compressions in the middle of the chest followed by 4 ventilations
□ 10 chest compressions in the middle of the chest followed by 4 ventilations

TURN OVER!

6: How do you perform artificial ventilation to an unconscious person?

□ As it is not included in basic life support, I will not do it
□ By blowing as much as I can into the mouth or nose, a bit like blowing up a balloon
□ Two breaths into the mouth or nose until the chest rises (correct)
□ By blowing rapidly into the person's mouth or nose until I can no longer breathe myself

7: What do you do if you are in doubt whether the person is having a cardiac arrest?

□ Wait for rescue workers to arrive, and then let them make the decision
□ Place the person in recovery position and wait for the assistance to arrive
□ Wait for five minutes and then try shaking the person again
□ If in doubt, I start basic life support (correct)

8: What is an AED?

□ I have no idea
□ An electronic device that can potentially restart the beating of the heart (correct)
□ An electronic device that only doctors, nurses and paramedics use in cardiac arrest situations
□ Something that belongs in a hospital

9: To what extent do you feel able to perform basic life support?

□ I am completely unable
□ I am very uncertain and would probably not be able to help
□ I know the theory but not what to do in practice
□ I feel well prepared and will take action if a person falls over

10: You witness a person suffering a cardiac arrest, and the person is in need of your help

Enter your fear on a scale of 0-10, with 10 being the worst imaginable fear:__________

Describe in one sentence what you fear the most about being the first person present at the scene of a cardiac arrest:
____

Thank you for your help!
